# A retrospective observational study of patients on maintenance hemodialysis receiving parathyroidectomy by ultrasonic scalpel

**DOI:** 10.1186/s12893-022-01634-8

**Published:** 2022-05-18

**Authors:** Dan Gao, Fengqi Hu, Zhao Gao, Hai Yuan

**Affiliations:** Department of Nephrology, Xiangyang Central Hospital, Hubei University of Arts and Science, Xiangyang, Hubei China

**Keywords:** Haemodialysis, Chronic kidney disease, Secondary hyperparathyroidism, Parathyroidectomy, Ultrasonic scalpel

## Abstract

**Background:**

Secondary hyperparathyroidism (SHPT) remains a common complication in many patients on maintenance hemodialysis. Kidney Disease Improve Global Outcomes (KDIGO) 2017 guidelines suggest that parathyroidectomy (PTX) should be performed in severe SHPT patients with chronic kidney disease stage 3a-stage 5D. In the present study, we observed the efficacy of ultrasonic scalpel for PTX in SHPT patients on maintenance hemodialysis.

**Methods:**

A total of 74 patients on maintenance hemodialysis who underwent PTX (34 with traditional electrocautery and 40 with an ultrasonic scalpel) were observed between August 2020 and August 2021 at Xiangyang Central Hospital (Hubei University of Arts and Science). Baseline demographic and clinic characteristics were collected pre- and post-PTX. Moreover, the postoperative complications and operation time were assessed between the two groups.

**Results:**

The univariate analysis showed that there was no statistical significance in weight, dialysis duration, serum potassium, serum calcium, serum magnesium, alkaline phosphate, triglyceride, and intact parathyroid hormone (iPTH) before and after PTX between the two groups (P > 0.05). The operation time in the ultrasonic scalpel group was significantly decreased compared with the traditional electrocautery group (P < 0.05). Compared with the traditional electrocautery group, the drainage amount was significantly reduced in the ultrasonic scalpel group, and the number of days with drain and postoperative hospital stay were also remarkably decreased (P < 0.05).

**Conclusions:**

The use of ultrasonic scalpel significantly reduced the operation time and postoperative hospital stay in patients on maintenance hemodialysis undergoing PTX.

## Introduction

Secondary hyperparathyroidism (SHPT) has a high prevalence in patients on maintenance hemodialysis. The prevalence of SHPT in maintenance HD patients is approximately 55–68.6% [1, 2]. The high level of parathyroid hormone (PTH) in SHPT patients is closely associated with mineral and bone disorders and mortality [3–5]. Although calcimimetics and vitamin D are widely used in SHPT patients on maintenance hemodialysis, some SHPT patients are refractory to medical treatments. Kidney Disease Improve Global Outcomes (KDIGO) 2017 guidelines suggest that parathyroidectomy (PTX) can be performed in severe SHPT patients with chronic kidney disease (CKD) stage 3a to stage 5D who fail to respond to medical therapy [6]. Therefore, PTX is required for SHPT patients who are resistant to medical therapy.

Bleeding is a common complication after thyroid or parathyroid operation [7, 8]. The traditional operations include blood vessel ligature and electrocoagulation. However, the operation time is prolonged, and the adjacent tissues are damaged due to thermal damage of electrocoagulation. Recently, an ultrasonic scalpel that employs mechanical vibration and can simultaneously cut and coagulate tissues has been used in several surgical operations [9, 10].

**Fig. 1:**
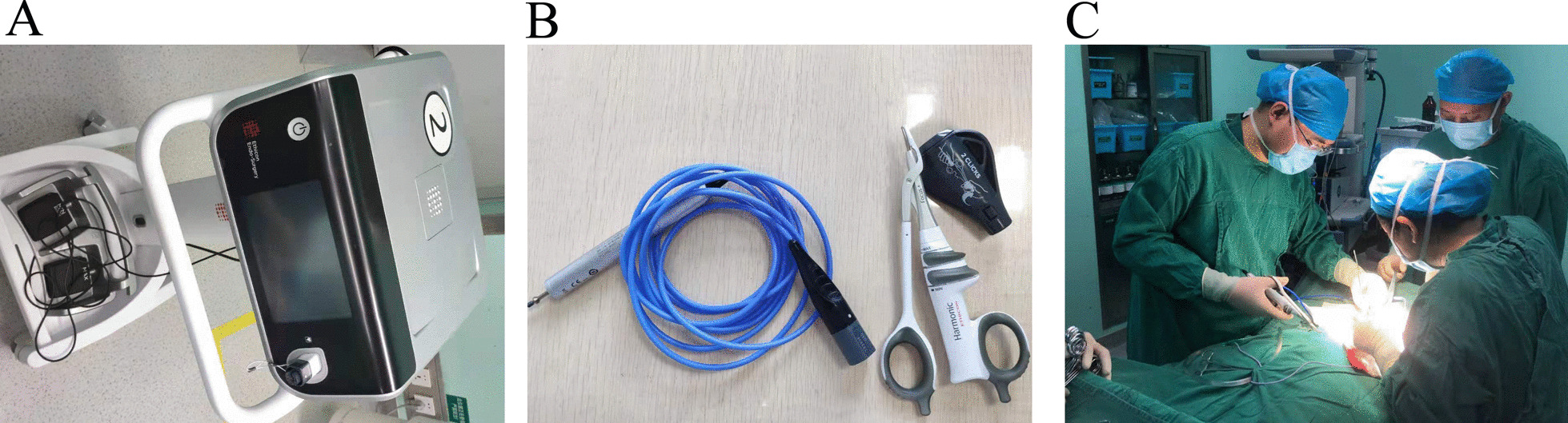
Ultrasonic scalpel is used in PTX. **A** and **B** Ultrasonic scalpel device, **C** Ultrasonic scalpel was used in operation

**Fig. 2:**
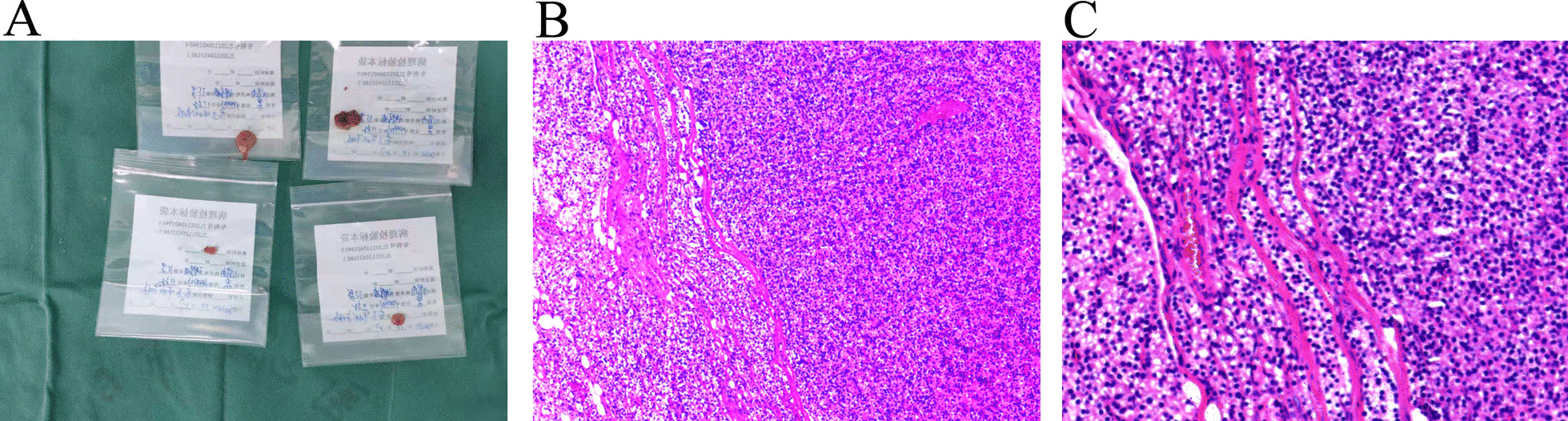
Parathyroid glands are analyzed by pathological examination. **A** Parathyroid glands were collected during operation. **B** Representative H&E staining of parathyroid glands. Original magnification × 100. **C** Representative H&E staining of parathyroid glands. Original magnification × 200

In the present study, we aimed to assess the value of ultrasonic scalpel in PTX. An ultrasonic scalpel was effectively and safely used in PTX (Fig. 1). The removed parathyroid glands could be confirmed by pathological examination (Fig. 2). The levels of PTH were usually declined to normal levels after PTX, indicating a successful operation. In addition, postoperative complications were observed between the electrocautery and ultrasonic scalpel groups.

## Materials and methods

### Patients

A total of 74 SHPT patients on maintenance hemodialysis were included in this analysis. Patients with heart failure, pulmonary insufficiency, or coagulation disorders were excluded. All patients had drug history of taking calcitriol, sevelamer and cinacalcet. PTX was performed in these patients because they failed to respond to medical treatment. Written informed consents were obtained from all participants.

### Surgery

A total of 34 patients underwent PTX by traditional electrocautery (ERBE, VIO 300D) from August 2020 to December 2020. Since January 2021, ultrasonic scalpels became available, and 40 patients received PTX using Harmonic Focus + Shears (Ultracision, Ethicon Endo-Surgery Europe). Four parathyroid glands were removed in all patients.

### Data collection

All patients’ age, sex, comorbidities, laboratory tests were collected. Biochemical analysis was tested by automatic biochemical analyzer (Siemens ADVIA2400). PTH was measured by chemiluminometric assay. The total alkaline phosphatase was detected.

### Statistical analysis

Continuous variables were presented as mean ± standard deviation and tested by a Mann–Whitney U-test and a Kruskal–Wallis test. Categorical variables were presented as frequency and tested by Pearson’s Chi-square test. The data were analyzed by SPSS 20.0. P values < 0.05 were considered statistically significant.

## Results

Table 1 show that the factors, such as median age, months on dialysis, body weight, and biochemical analysis, in both groups of patients were not significantly different before PTX. The levels of serum phosphorus, serum calcium, and intact parathyroid hormone (iPTH) in both groups were significantly decreased after PTX. The level of alkaline phosphate in both groups was significantly increased after PTX. However, there was no difference in terms of serum phosphorus, serum calcium, serum magnesium, iPTH, and alkaline phosphate between the two groups after PTX (Table 2).

**Table 1:** Demographic and clinic characteristics of patients before PTX

	Electrocautery (34)	Ultrasonic scalpel (40)	P-value
Age, mean (SD), years	48.68 ± 11.15	50.92 ± 16.17	0.645
Months on dialysis, mean (SD)	98.79 ± 38.99	85.62 ± 26.98	0.373
Female/male, n (%)	16 (47.1%)	21 (52.5%)	0.816
Body weight (kg)	66.00 ± 10.23	69.09 ± 10.94	0.498
Diabetes mellitus, n (%)	6 (17.6%)	9 (22.5%)	0.775
Hypertension, n (%)	25 (73.5%)	32 (80.0%)	0.585
Hepatitis virus, n (%)	4 (16.7%)	6 (15.0%)	0.745
Serum potassium (mmol/L)	4.24 ± 0.32	4.36 ± 0.42	0.675
Serum calcium (mmol/L)	2.48 ± 0.29	2.33 ± 0.19	0.105
Alkaline phosphate (U/L)	251.00 (128.00, 526.00)	402.00 (146.50, 792.50)	0.377
Triglyceride (mmol/L)	2.50 ± 0.32	2.13 ± 0.56	0.317
Total cholesterol (mmol/L)	4.19 ± 0.83	3.87 ± 1.15	0.228
Albumin (g/L)	42.35 ± 5.21	39.26 ± 4.00	0.082
iPTH, pg/mL	1433.93 ± 812.04	1366.00 ± 747.26	0.886
Serum phosphorus (mmol/L)	2.33 ± 0.66	2.48 ± 0.68	0.464
Serum magnesium (mmol/L)	0.98 ± 0.12	0.99 ± 0.14	0.844

**Table 2:** Demographic and clinic characteristics of patients after PTX

	Electrocautery (34)	Ultrasonic scalpel (40)	P-value
Serum phosphorus (mmol/L)	1.80 ± 0.46	1.84 ± 0.38	0.759
Serum calcium (mmol/L)	2.37 ± 0.20	2.24 ± 0.22	0.096
Serum magnesium (mmol/L)	0.95 ± 0.09	0.93 ± 0.10	0.588
Alkaline phosphate (mmol/L)	517.50 (168.42, 897.54)	585.70 (148.75, 924.23)	0.564
iPTH (pg/mL)	82.55 (29.35, 172.88)	58.00 (28.25, 182.60)	0.873

Operation time and drainage amount were significantly decreased in the ultrasonic scalpel group compared with the electrocautery group (94.00 ± 16.84 min vs. 123.00 ± 25.16 min; 119.31 ± 51.77 ml vs. 145.58 ± 46.83 ml, respectively). Moreover, the number of days with drain and postoperative hospital stay were significantly reduced in the ultrasonic scalpel group compared with the electrocautery group (4.40 ± 1.62 days vs. 5.80 ± 2.14 days; 8.60 ± 3.65 days vs. 10.70 ± 4.21 days, respectively). In addition, there was no difference in postoperative complications between the two groups (Table 3).

**Table 3:** Comparison between patients operated with electrocautery and ultrasonic scalpel

	Electrocautery (34)	Ultrasonic scalpel (40)	P-value
Operating time (min)	123.00 ± 25.16	94.00 ± 16.84	0.030
Drainage amount (mL)	145.58 ± 46.83	119.31 ± 51.77	0.020
Recurrent laryngeal nerve injury	1 (2.9%)	0 (0%)	0.460
Postoperative infection	1 (2.9%)	0 (0%)	0.460
Number of days with drain	5.80 ± 2.14	4.40 ± 1.62	0.042
Postoperative hospital stay (days)	10.70 ± 4.21	8.60 ± 3.65	0.030

## Discussion

Parathyroid glands are usually located on the back of the thyroid and near the recurrent laryngeal nerve. To remove parathyroid glands, the thyroid must be turned up. Postoperative bleeding is usually a common complication because the thyroid is highly vascularized. In addition, serious postoperative bleeding is life-threatening because of neck hematoma [7]. Effective intraoperative hemostasis is important in neck surgery. The common complications of PTX are bleeding, infection, and hypocalcemia [11, 12]. To decrease the frequency of surgical complications, we observed the effects of traditional electrocautery and ultrasonic scalpels in this study.

The results of our study demonstrated that the operation time and drainage amount were significantly decreased in the ultrasonic scalpel group compared with the electrocautery group. It is well known that ultrasonic scalpel transforms electric energy into mechanical vibration. An ultrasonic scalpel can lead to vaporization, protein coaptation, and protein denaturation at a temperature lower than 100 °C. However, electrocautery leads to the carbonization of tissue and reaches temperatures as high as 400 °C [13]. In addition, the number of days with drain and postoperative hospital stay were shorter in the ultrasonic scalpel group. Therefore, an ultrasonic scalpel could decrease the operation time and complications. The incidence of recurrent laryngeal nerve injury and postoperative infection in the electrocautery group was higher compared with the ultrasonic scalpel group. However, there were no significant differences between the two groups. Obviously, tissue damage was less in ultrasonic scalpel operation because of lower temperature and shorter operation time compared with the traditional operation.

Bipolar and monopolar cautery, vascular ligations, and hemostatic clips are used for hemostasis in conventional operation. These conventional techniques are often considered reliable methods for intraoperative hemostasis. However, bipolar electrocautery has been used only for very small vessels, and vascular ligation and hemostatic clips are very time-consuming techniques. Ultrasound is a more recently developed hemostatic technique with high efficacy. Ultrasonic vessel sealing devices have been shown to reduce operation time and a variety of postoperative complications, such as thermal nerve injury during thyroidectomy [14].

Our study had several limitations. First, our study was not a randomized controlled study. Second, the sample size of this study was small. Therefore, large-scale randomized controlled studies are required in the future.

In conclusions, an ultrasonic scalpel was effective and safe in PTX. The use of ultrasonic scalpel significantly reduced the operation time and postoperative hospital stay in patients on maintenance hemodialysis undergoing PTX.

## Data Availability

The datasets used and analyzed during the current study are available from the corresponding author on reasonable request.
